# The Platelet Activation Signaling Pathway Regulated by Fibrinogen and Homo-Gamma-Linolenic Acid (C20:3)-Associated Lipid Metabolism Is Involved in the Maintenance of Early Pregnancy in Chinese Native Yellow Cattle

**DOI:** 10.3390/ani15091219

**Published:** 2025-04-25

**Authors:** Miao Yu, Changzheng Du, Yabo Ma, Yuqin Ma, Pengfei Li, Xianguo Xie, Mengyuan Li, Xueyi Nie, Yueyang Liu, Yuxin Hou, Shenao Miao, Xingping Wang, Jinrui Xu, Yi Yang

**Affiliations:** 1 School of Life Sciences, Ningxia University, Yinchuan 750021, China; klimsman@163.com (M.Y.); duchangzheng123@outlook.com (C.D.); myb512816@163.com (Y.M.); 15909611973@163.com (Y.M.); lpflipengfei123@outlook.com (P.L.); 18309570750@139.com (X.X.); 20180044@nxmu.edu.cn (M.L.); 18794898774@163.com (X.N.); 18395273708@163.com (Y.L.); 18209689779@163.com (Y.H.); msa317169@163.com (S.M.); wxp@nxu.edu.cn (X.W.); 2 Key Laboratory of Ministry of Education for Conservation and Utilization of Special Biological Resources in the Western, Ningxia University, Yinchuan 750021, China; 3 College of Animal Science and Technology, Ningxia University, Yinchuan 750021, China

**Keywords:** Chinese native yellow cattle, early pregnancy, metabolism, immune response, angiogenesis

## Abstract

Identifying the specific factors secreted during early pregnancy is an effective method for pregnancy detection in cattle, helping to reduce empty pregnancies in the industry. To systematically investigate metabolic variations between early pregnancy and the estrous cycle and their relationship with pregnancy progression, this study utilized four-dimensional data-independent acquisition (4D-DIA) proteomics and liquid chromatography–tandem mass spectrometry (LC-MS/MS) metabolomics to analyze serum samples collected from Chinese native yellow cattle at day 0 and day 21 post-mating, combining bioinformatics analysis with experimental validation. The platelet activation signaling pathway and angiogenesis-related proteins were significantly upregulated. Among them, fibrinogen alpha/beta/gamma chains (FG) exhibited notable differences, with their branched-chain protein FGB showing highly significant upregulation (*p* = 0.003, Log_2_FC = 2.167) and tending to increase gradually during early pregnancy, suggesting that FGB could be one of the important indicators of early pregnancy in Chinese native yellow cattle. Among the differential metabolites, 11-Deoxy prostaglandin F1α (*p* < 0.001, Log_2_FC = 1.563), Thromboxane B1 (*p* = 0.002, Log_2_FC = 3.335), and Homo-Gamma-Linolenic Acid (C20:3) (*p* = 0.018, Log_2_FC = 1.781) were also increased, indicating their involvement in the regulation of the platelet activation signaling pathway. The platelet activation signaling pathway plays a crucial role in maternal immune tolerance and placental vascularization, which are essential for embryo implantation and placental development. These findings indicate that FGB has the potential to be a valuable biomarker for early cattle pregnancy detection, thereby improving pregnancy diagnosis accuracy, reducing economic losses caused by undetected empty pregnancies and enhancing reproductive efficiency in the cattle industry. Undoubtedly, our research outcomes must be validated with future studies, and a larger sample size as well as the evaluation of the potential endocrine effects induced by the synchronized estrus treatment must be considered.

## Simple Summary

Early pregnancy detection in cattle is critical for improving reproductive efficiency and reducing economic losses. This study investigated changes in blood proteins and metabolites in Chinese native yellow cattle during early pregnancy using advanced proteomic and metabolomic techniques. Blood samples were collected from pregnant and non-pregnant cows at day 0 and day 21 post-mating. Our results revealed the significant upregulation of the fibrinogen beta chain (FGB) and metabolic changes related to platelet activation and vascular development. These findings suggest that FGB could serve as a novel biomarker for early pregnancy detection, providing a valuable tool for improving cattle breeding management and productivity.

## 1. Introduction

Guyuan Yellow Cattle, a distinct breed indigenous to the Ningxia Hui Autonomous Region of China, is highly valued for its meat quality and has been selectively bred under the region’s unique ecological conditions. This breed originated from the crossbreeding of Mongolian cattle [1] and Qinchuan cattle [2] and has developed a strong adaptability and high reproductive efficiency. Studying early pregnancy biomarkers in Guyuan Yellow Cattle is essential for optimizing breeding management and enhancing reproductive success in regional livestock production. This experiment explores the differences in serum metabolites between early pregnancy and the end of estrus in this type of cow, as well as the relationships among these metabolites, and further investigates the mechanism for maintaining pregnancy physiology. Estrus detection [3] and early pregnancy diagnosis [4] in dairy cows are essential tools in bovine reproductive management. The former allows for accurate prediction of ovulation, while the latter plays a crucial role in enhancing reproductive efficiency and economic sustainability in the livestock industry. Advances in biomarker-based diagnostic methods enable the more precise identification of non-pregnant cows, helping to reduce reproductive losses and improve farm profitability [5].

Specific proteins and endogenous hormones secreted during early pregnancy are essential for pregnancy maintenance [4]. These substances are secreted into body fluids such as milk, blood, and urine during pregnancy [6]. However, at the early stage, the cows have a high rate of pregnancy loss. After fertilization, the early embryo secretes special factors to establish initial contact with the mother. If the quality of the fertilized egg is not up to standard, or if the mother fails to reach the gestational state, it results in a 90% loss of early embryos [7,8,9,10,11]. As the gestational state tends to be stabilized, the accuracy of diagnosis using the special protein of early pregnancy reaches over 95% around the 28th day. This time point is widely used for early biomarker-based pregnancy detection in cattle and is notable for its simplicity from a practical standpoint [4].

The bovine estrous cycle lasts approximately 21 days, making it a critical reference point for pregnancy diagnosis. However, the 21st day of fertilization is an important stage that is still being tackled by today’s technology for early cattle pregnancy detection [12]. Currently, the analysis of protein and metabolic components in early pregnancy cows is groundbreaking in the search for biological markers and physiological changes in early pregnancy [13]. A comparative study of the serum protein content of Holstein cows from the end of estrus to the 21st day of pregnancy using LC-MS/MS technology revealed the differential expression of APOB, SPADH1, PLIN2, and LPO proteins (*p* < 0.05) [14]. Additionally, Bahuguna, C. analyzed serum samples from Sahiwal cows on day 19 of pregnancy and non-pregnancy, identifying lactotransferrin, Golgin A4, MYRIP, PKD1, and the PWWP domain-containing protein MUM1 as potential biomarkers for pregnancy diagnosis. These proteins were detected using nano-LC–MS/MS following trypsin digestion. Although this method necessitates specialized equipment, reagents, and trained personnel, leading to higher costs, its high sensitivity, resolution, and throughput offer substantial benefits for protein identification and quantification, making it cost-effective for large-scale pregnancy biomarker analysis [15].

In this experiment, we utilized four-dimensional data-independent acquisition (4D-DIA) and liquid chromatography–tandem mass spectrometry (LC-MS/MS) to investigate serum protein and metabolic profiles in Chinese native yellow cattle. The 4D-DIA method is an advanced proteomic technique that enhances sensitivity and quantification accuracy by incorporating ion mobility separation, allowing for the precise identification of differentially expressed proteins. LC-MS/MS enables high-throughput metabolite profiling, providing comprehensive insights into metabolic changes. Serum samples were collected from the same pregnant cows on day 0 and day 21 after mating to analyze changes in protein and metabolite levels, assess associations between differentially expressed proteins and metabolites, identify a potential biomarker for early pregnancy detection, and explore the mechanisms contributing to pregnancy stability during early gestation.

## 2. Materials and Methods

### 2.1. Animal Preparation

This study was approved by the Animal Experimental Ethics Committee of Ningxia University (Approval No.: NXU-22-015).

The experimental site was chosen as the Fumin Agricultural Yellow Cattle Breeding Base in Yuanzhou District, Guyuan City, China. A large number of local high-quality yellow cattle were sourced from local vendors and were then serologically tested for population identification and to exclude unsuitable individuals. A total of 37 yellow cattle were initially identified, and 30 of them underwent ultrasound examination. Among these, nine cows were confirmed as non-pregnant and were selected for further experiments. The cattle were screened based on their health status and reproductive history, with individuals exhibiting reproductive disorders or poor health being excluded to ensure the reliability of the study (Figure S1A).

### 2.2. Simultaneous Estrus and Natural Mating in Non-Pregnant Cows

The selected nine cows received sequential hormonal treatments: (1) 200 μg gonadorelin (intramuscular) in the morning of day 1, (2) 1 mg cloprostenol sodium (intramuscular) in the morning of day 7, and finally (3) 200 μg gonadorelin in the afternoon of day 9. Natural mating with fertile bulls was conducted in the morning of day 10, attributable to the absence of frozen semen banks for indigenous cattle breeds. All the injection reagents were sourced from (Sansheng Biotechnology, Ningbo, China) (Figure S1B).

### 2.3. Sample Collection and Selection

Serum samples were obtained at 0, 7, 14, 21, 28, and 35 days post-mating through standardized venipuncture procedures. Following each collection, jugular venous blood was drawn into 15 mL anticoagulant-free vacuum tubes, allowed to clot at 4 °C for 30 min, then centrifuged at 3000× *g* for 15 min (4 °C) to separate the serum fractions. The supernatant was aliquoted into pre-chilled 1.8 mL cryovials and immediately flash-frozen in liquid nitrogen for long-term preservation at −80 °C (Figure S1C).

On day 28 after mating, a cattle early pregnancy test was conducted on the nine cows using the Bovine Cow Rapid Early Pregnancy Test Kit (Fende Biotech, Shenzhen, China) with blood samples collected from the coccygeal vein, which diagnosed six cows as pregnant and three cows as non-pregnant. Ultrasound examinations performed on day 35 after mating confirmed these results (Figure S1D,E).

Serum samples were taken on day 0 and day 21 after mating, from which three pregnant cows and three non-pregnant cows were randomly selected for 4D-DIA proteomic analysis and LC-MS/MS metabolite analysis.

### 2.4. Sample Analysis

The cryopreserved serum samples from Section 2.3 were separated into pregnant day 0, non-pregnant day 0, pregnant day 21, and non-pregnant day 21 groups (*n* = 3). Novogene Co., Ltd. (Beijing China) performed 4D-DIA proteomic analysis and LC-MS/MS metabolite analysis. Detailed sample processing, instrument parameter settings, and data acquisition and analysis can be found in the Supplementary Materials. 

### 2.5. Validation

Albumin was removed from serum using an albumin removal kit (Proteintech, Chicago, IL, USA).

Western blot analysis was performed on blood samples from all nine cattle (six pregnant, three non-pregnant) on days 0 and 21 post-mating. Each sample was analyzed in triplicate for technical validation. The specific experimental procedures were as follows:

The protein concentration was determined using a BCA assay (KeyGen Biotech, Nanjing, China). For each sample, 30 μg of protein was loaded onto an SDS-PAGE gel for electrophoresis, followed by wet transfer onto a nitrocellulose (NC) membrane (Millipore, Billerica, MA, USA). Ponceau S staining (Solarbio, Beijing, China) was used to confirm uniform transfer and equal sample loading. The membrane was incubated with primary antibodies against fibrinogen (GeneTex, Irvine, CA, USA), FGB (Affinity Biosciences, Changzhou, China), and complement component 4 binding protein alpha (C4BPA) (Affinity Biosciences, Changzhou, China), followed by a peroxidase-conjugated secondary antibody (ZSGB-BIO, Beijing, China). The protein bands were visualized using an enhanced chemiluminescence detection kit (Affinity Biosciences, Changzhou, China).

Further Western blotting to detect the FGB protein was performed in the serum of pregnant and non-pregnant cows at different stages (0, 7, 14, 21, 28, and 35 days after mating). The serum samples were obtained from blood collection as described in Section 2.3. From the serum of all nine cows, three pregnant and three non-pregnant cows were randomly selected to represent the pregnant and non-pregnant groups for the Western blotting analysis of FGB protein expression. The specific experimental procedures for Western blotting were the same as those described in the previous section.

To verify the metabolomics results and minimize individual variability, we expanded LC-MS/MS validation to five biological replicates per group, incorporating the day 28 samples, with all samples prepared as pooled mixtures by averaging the individual samples within each group.

### 2.6. Statistical Analysis

All Western blotting experiments were performed with three technical replicates, and the data are presented as the mean ± SEM. Protein absorbance (A) values were analyzed using the ImageJ 1.54f software, and statistical analyses were conducted using One-way ANOVA or T-tests in the GraphPad Prism 8.0 software. Differences were considered significant at *p* < 0.05.

## 3. Results

### 3.1. Differential Protein Analysis

Using 4D-DIA proteomics, we identified 355 serum proteins in early pregnancy samples. Differential expression analysis, applying thresholds of |log_2_FC| > 0.263 (equivalent to FC > 1.2 or <0.833) and statistical significance (*p* < 0.05), revealed fourteen differentially expressed proteins (DEPs), comprising six upregulated and eight downregulated candidates (Table S1).

**Figure 1:**
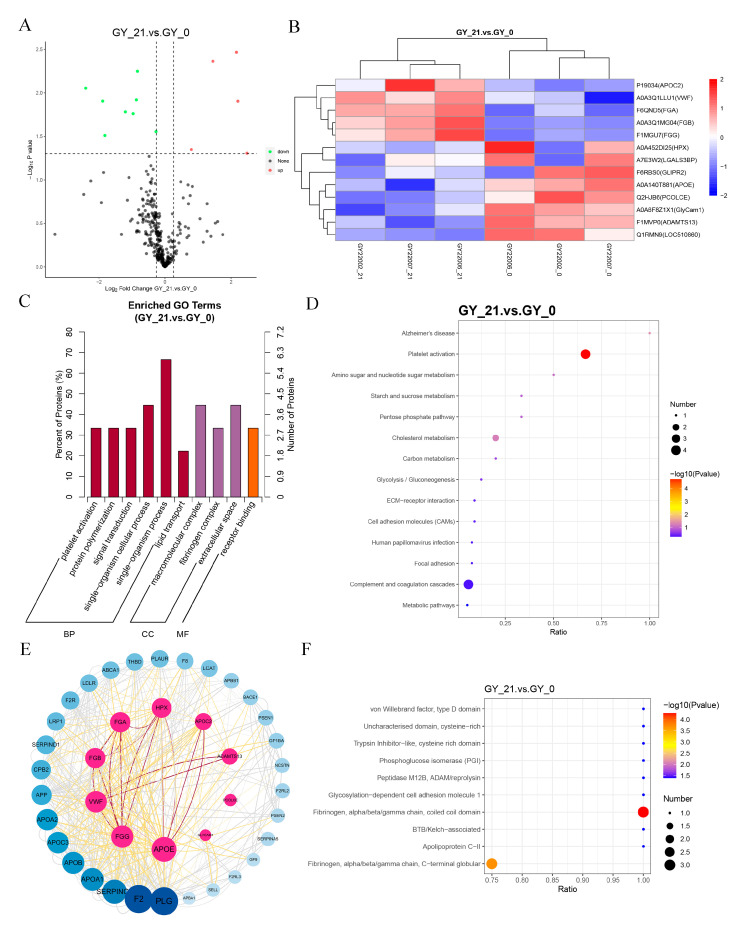
Differential protein analysis of early pregnancy serum from cattle. (**A**) Volcano plot of differentially expressed proteins (DEPs) in early pregnancy. The *x*-axis represents fold changes (log_2_FC), while the *y*-axis indicates statistical significance (-log_10_ *p*-value). The red and green dots denote significantly upregulated and downregulated proteins, respectively, while black dots represent non-significant proteins. (**B**) Hierarchical clustering heatmap of DEPs. The *x*-axis represents sample clustering, and the *y*-axis represents protein clustering, with shorter branches indicating a higher similarity. (**C**) GO enrichment analysis of DEPs, categorizing proteins based on biological processes, molecular functions, and cellular components. The *x*-axis represents GO terms, while the *y*-axis indicates the proportion of enriched DEPs relative to the total DEPs. (**D**) KEGG pathway enrichment analysis of DEPs, highlighting the most significantly affected pathways in early pregnancy. The *x*-axis represents the proportion of DEPs within each pathway, while color intensity and bubble size indicate the *p*-values and the number of proteins involved. (**E**) Protein–protein interaction (PPI) network analysis. Each node represents a protein, with inner circles indicating DEPs and outer circles denoting functionally related proteins. Node size and color intensity reflect the degree of interaction. (**F**) IPR (InterPro) structural domain enrichment analysis, identifying the key protein domains associated with pregnancy. The *x*-axis represents the proportion of enriched DEPs, with color and size indicating statistical significance.

A volcano plot visualization highlighted the distinct expression patterns of DEPs (Figure 1A), while hierarchical clustering demonstrated the strong intra-group consistency and inter-group divergence (Figure 1B). Gene Ontology (GO) enrichment categorized DEPs primarily into platelet activation, lipid metabolism, and single-organism metabolic processes (Figure 1C).

KEGG pathway analysis identified two pregnancy-critical pathways: platelet activation signaling and complement/coagulation cascades (Figure 1D). Protein–protein interaction (PPI) networks further underscored the centrality of coagulation-related proteins (Figure 1E). Structural domain (IPR) analysis specifically implicated fibrinogen α/β/γ chains, with the β subunit (FGB) exhibiting significant differential expression (*p* = 0.003, log_2_FC = 2.167) (Table S1). The progressive elevation of FGB during early gestation suggested its potential as a promising diagnostic biomarker.

A comprehensive functional annotation of all DEPs was compiled to elucidate their roles in gestational physiology (Table S2).

### 3.2. Differential Metabolite Analysis

To complement the proteomic findings, untargeted metabolomic profiling via LC-MS/MS identified 723 serum metabolites. Applying multivariate criteria (VIP > 1.0, |log_2_FC| > 0.263, *p* < 0.05), we detected 53 differentially abundant metabolites (DAMs), including 31 upregulated and 22 downregulated species (Table S3).

**Figure 2:**
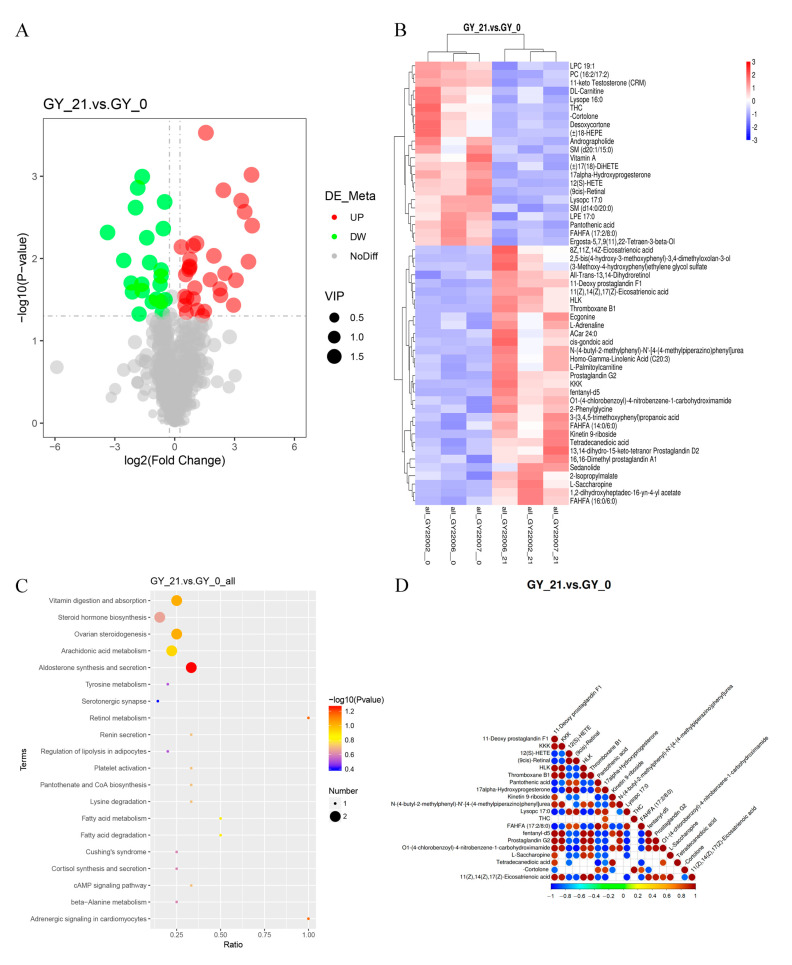
Differential metabolite analysis in the early pregnancy serum of cattle. (**A**) Volcano plot of differentially abundant metabolites (DAMs). The *x*-axis represents fold changes (log_2_FC), while the *y*-axis denotes statistical significance (-log_10_ *p*-value). The red and green dots indicate significantly upregulated and downregulated metabolites, respectively, while the black dots represent non-significant metabolites. The size of each dot corresponds to the VIP (variable importance in projection) score. (**B**) Hierarchical clustering heatmap of DAMs, illustrating the metabolic differences between pregnant and non-pregnant cattle. The *x*-axis represents sample clustering, and the *y*-axis represents metabolite clustering, with shorter branches indicating higher similarity. (**C**) KEGG pathway enrichment analysis of DAMs, identifying the key metabolic pathways involved in early pregnancy. The *x*-axis represents the proportion of DAMs within each pathway, while bubble color and size indicate statistical significance and the number of metabolites involved. (**D**) Correlation heatmap of differential proteins and metabolites, showing the relationships between DAMs and DEPs. The color scale represents the correlation coefficients, with red indicating positive correlations and blue denoting negative correlations. *p*-values < 0.05 were considered significant.

Volcano plot analysis resolved the DAM distribution patterns, highlighting prostaglandin derivatives and fatty acids as dominant upregulated classes (Figure 2A). Hierarchical clustering revealed a clear segregation between pregnant and non-pregnant cohorts, with tight intra-group metabolite correlations (Figure 2B). KEGG pathway enrichment implicated ovarian steroidogenesis and arachidonic acid metabolism as central regulatory axes (Figure 2C). The top 20 significant DAMs demonstrated functional convergence in three key gestational processes: inflammatory modulation (11-Deoxy prostaglandin F1α); Vasoregulation (Thromboxane B1); and angiogenic priming (Homo-γ-Linolenic acid).

Strikingly, these lipid mediators exhibited strong positive correlations with the FGB levels, mechanistically linking platelet activation signaling from proteomic data to metabolic regulation (Figure 2D). A systematic functional annotation of DAMs further contextualized their roles in early gestational adaptation (Table S4). 

### 3.3. Association Analysis of Differential Proteins and Differential Metabolites

**Figure 3:**
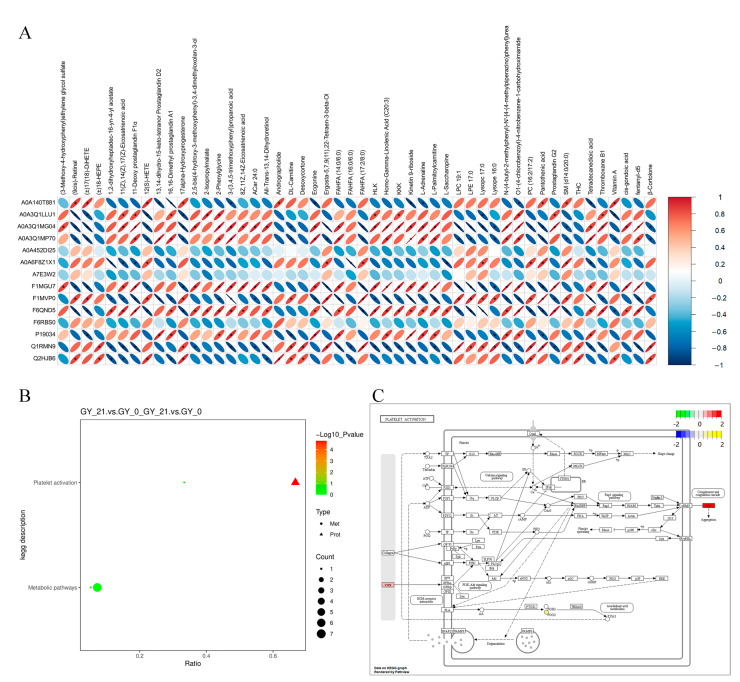
Multi-omics integration of differential proteins and metabolites. (**A**) Heatmap of the correlation analysis between DEPs and DAMs. The *x*-axis lists DEPs, and the *y*-axis lists DAMs. Red represents positive correlations, while blue indicates negative correlations. The asterisk (*) indicates statistical significance, defined as *p* < 0.05. (**B**) KEGG co-enrichment analysis, identifying the pathways jointly enriched in proteomic and metabolomic datasets. The *x*-axis represents the proportion of enriched DEPs and DAMs, with bubble color and size indicating statistical significance and the number of molecules involved. (**C**) KEGG pathway visualization illustrating metabolite and protein interactions within the enriched pathways. Nodes are colored based on log_2_FC values, with proteins represented in a green-to-red gradient and metabolites in a blue-to-yellow gradient.

The integrated multi-omics approach enabled cross-validation between proteomic and metabolomic datasets, enhancing the biological relevance of our findings. Spearman correlation analysis revealed the coordinated regulation between DEPs and DAMs, exhibiting both positive and negative associations across gestational states (Figure 3A). KEGG co-enrichment analysis identified the platelet activation pathway as the most statistically significant node, with FG demonstrating particularly strong metabolite covariation (Figure 3B).

Notably, the FGB showed the highest network centrality and metabolite correlation density, forming functional clusters with key lipid mediators including 11-Deoxy prostaglandin F1α and Homo-γ-Linolenic acid (Figure 3C). This tight coordination between coagulation proteins and inflammatory lipid metabolites underscores FGB’s dual role as both a mechanistic driver and a potential diagnostic marker for early bovine pregnancy.

### 3.4. Validation

**Figure 4:**
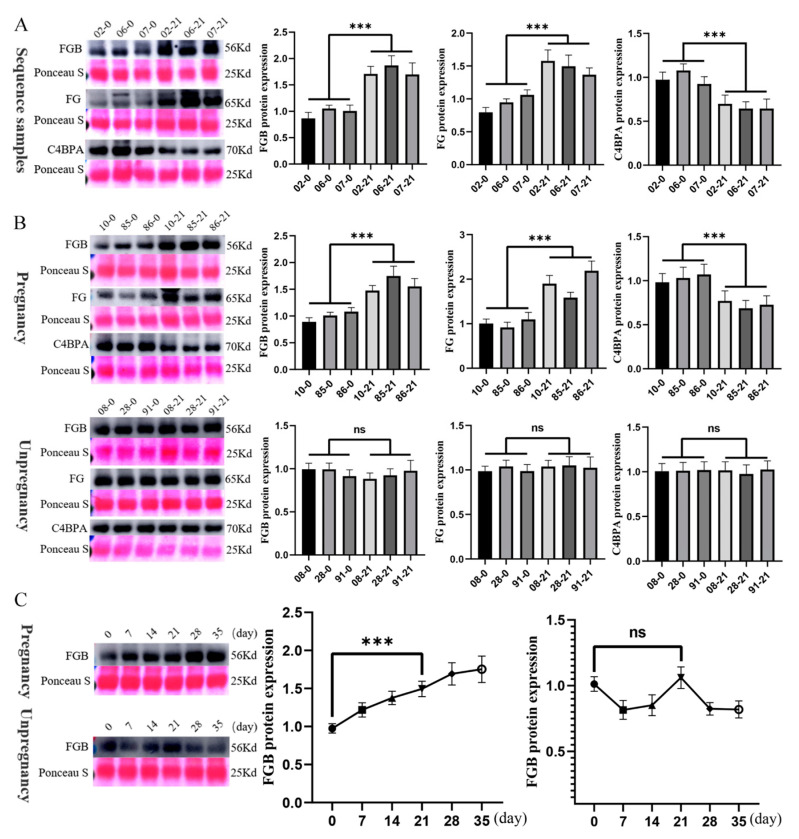
Western blot validation of differentially expressed proteins in early pregnancy. (**A**) Western blot validation of the 4D-DIA proteomic findings. Serum samples from pregnant and non-pregnant cattle (collected at days 0 and 21 post-mating) were analyzed to verify the differential protein expression. Representative immunoblot bands and corresponding quantifications are shown (*n* = 3 per group). The numbers before the hyphen represent the sample ID, while the numbers after the hyphen indicate the days of pregnancy. (**B**) Western blot validation of FGB expression in an independent cohort. Serum samples from three pregnant and three non-pregnant cattle were assessed to confirm the diagnostic consistency of FGB at early pregnancy time points (days 0 and 21). (**C**) Longitudinal profiling of FGB expression during early pregnancy. Western blot analysis of serum samples from pregnant and non-pregnant cattle across multiple time points (days 0, 7, 14, 21, 28, and 35 post-mating) revealed a progressive increase in FGB levels in the pregnant group. In contrast, FGB expression remained relatively stable in non-pregnant cattle. Data are presented as mean ± SEM, with *** *p* < 0.001 indicating statistical significance; ns = not significant.

To validate the proteomic findings, we prioritized three candidate proteins that exhibited both statistical significance (*p* < 0.01) and substantial fold changes (log_2_FC > 2.0): FGB, C4BPA, and FG. Western blot validation in the original cohort demonstrated a strong concordance with mass spectrometry data (Figure 4A). Furthermore, replication in an independent cohort (three pregnant vs. three non-pregnant cattle) confirmed the diagnostic consistency of FGB (Figure 4B).

Longitudinal profiling revealed the progressive accumulation of FGB across gestational timepoints (days 0–35). In contrast to the non-pregnant group in the right panel, where FGB levels remained relatively stable, the pregnant group in the left panel exhibited a sustained elevation throughout the observation period (Figure 4C).

**Figure 5:**
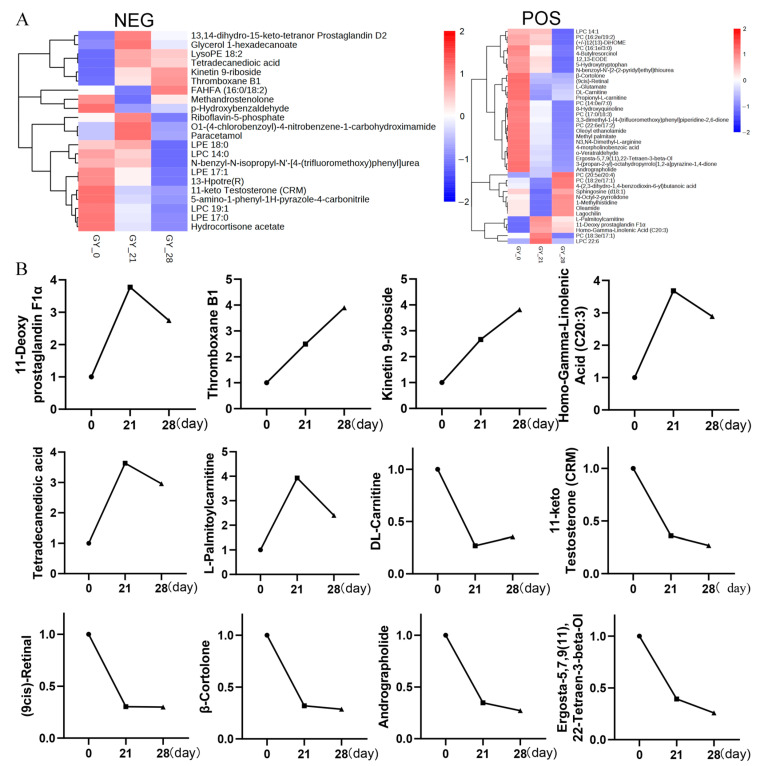
Metabolomic validation and temporal dynamics of key differential metabolites. (**A**) Hierarchical clustering heatmap of DAMs. Pregnant and non-pregnant cattle exhibit distinct metabolic profiles, with strong intra-group consistency and clear inter-group differences. (**B**) Temporal trends of key platelet-associated metabolites. Line graphs depict the relative abundance of 11-Deoxy prostaglandin F1α, Thromboxane B1, and Homo-γ-Linolenic acid across the early pregnancy time points (days 0, 21, and 28 post-mating). Thromboxane B1 shows a continuous increase from day 0 to day 28, whereas 11-Deoxy prostaglandin F1α and Homo-γ-Linolenic acid exhibit a sharp rise at day 28 followed by a slight decline. Data were derived from LC-MS/MS analysis and are presented as mean ± SEM.

To validate the metabolomic results and reduce variability, we analyzed five pooled intra-group specimens per group (*n* = 5/group) via LC-MS/MS, including the day 28 samples. The clustering heatmap of differentially abundant positive and negative ion metabolites between pregnant and non-pregnant samples further validated our findings (Figure 5A). This enhanced design confirmed the stable upregulation of platelet-associated metabolites—11-Deoxy prostaglandin F1α, Thromboxane B1, and Homo-γ-Linolenic acid—during early pregnancy. Notably, Thromboxane B1 exhibited a continuous increase at days 0, 21, and 28, whereas 11-Deoxy prostaglandin F1α and Homo-γ-Linolenic acid showed a marked surge at day 28, followed by a slight decline. The line graph in Figure 5B, derived from a statistical analysis of the differential metabolites identified via LC-MS/MS metabolomics, clearly illustrates these trends.

Collectively, these findings suggest that the coordinated activation of platelet signaling pathways—particularly their roles in angiogenesis and maternal immune modulation—emerges as a crucial regulatory axis warranting further investigation in the context of bovine early pregnancy physiology (Figure 5 and Figure S4).

## 4. Discussion

### 4.1. Innovativeness and Feasibility of the Experiment

The integration of the 4D-DIA and LC-MS/MS technologies demonstrates distinct analytical advantages over conventional methods, particularly in measurement precision, quantitative accuracy, experimental comprehensiveness, and processing throughput. This investigation systematically combines these emerging high-resolution proteomic platforms with well-established Western blotting methodology to identify early pregnancy biomarkers and characterize the metabolic dynamics in Chinese indigenous yellow cattle at gestational day 21. The selected time point corresponds to a critical phase in bovine reproductive biology, coinciding with both the putative window for early pregnancy confirmation and the typical estrous cycle duration [4,12]. This strategic methodological synergy ensures the reliability and discriminative power of experimental outcomes through complementary validation approaches.

### 4.2. Analysis of Physiological Changes in Early Pregnancy in Cattle by Identified Proteins and Metabolites

This study utilized 4D-DIA technology to identify and annotate pregnancy-related differential proteins, aiming to investigate the physiological changes in cows before and after pregnancy. Through searching different databases, it was found that the metabolism of cows changed dramatically during early pregnancy, the immune system and endocrine system were highly activated due to the active defense mechanisms in the body, and the changes in the digestive system might have been the direct cause of the metabolic changes (Figure S2). Therefore, we conducted a metabolic profiling analysis of serum samples from cows at day 0 and day 21 after mating using LC-MS/MS technology. Then, by annotating their functions and classifications, we found that endocrine system secretion and the digestive system related to metabolism were very active in the early pregnancy period of cattle. Lipid metabolism was the most intense in this case, consistent with proteomics results, which provided strong support for an active single-organism process and altered steroid hormone secretion in the endocrine system (Figure S3). In summary, the analysis of the proteins and metabolites identified suggests that the digestive system is dramatically altered during early gestation in cattle, with changes in maternal intestinal permeability accompanied by the accelerated absorption of nutrients such as proteins and lipids, resulting in a high level of metabolic activity in the cows, including amino acid metabolism and lipid metabolism [16,17]. The active metabolism provides the basis for the activation of the immune and endocrine systems, which in turn promotes biological processes such as inflammation response, immune response, and steroid hormone synthesis [17], and also provides the energy and material basis for single-organism processes [18].

### 4.3. Differential Proteins and Differential Metabolites to Analyze Physiological Changes in Early Pregnancy in Cattle

In differential protein and differential metabolite analysis, the platelet activation signaling pathway was found to be highly significant, in which the FG protein plays an important role in immune response and coagulation [16,19]. The levels of this protein have been observed to increase during gestation in both humans and cattle, indicating a potential biological consistency across species [20,21]. Compared to Holstein cows, the upregulation of FGB in early pregnancy in Chinese native yellow cattle was more pronounced, possibly due to breed-specific genetic factors and reproductive adaptations; VWF and ADAMTS13 mediated platelet adhesion and aggregation, which was important in promoting angiogenesis [16,22,23]; maternal hemoglobin was absorbed by the placenta for embryonic vascular development and fetal growth [24,25]; and the differential expression of these proteins marked the activation of the immune system and the establishment of placental tissues during early gestation (Tables S1 and S2). In addition, the differential expression of lipid metabolism is prominent during pregnancy. Specifically, APOC2 and APOE regulate lipid transport, cholesterol metabolism, and immunomodulation [17,26]. During pregnancy, cows absorb substantial lipids and bioactive compounds to enhance metabolic demands. Linolenic acid—an essential fatty acid requiring dietary intake—serves as the primary precursor for arachidonic acid synthesis via linoleic acid conversion. Subsequent metabolites, including 11-Deoxy prostaglandin F1α, Prostaglandin G, Thromboxane B1, Homo-Gamma-Linolenic Acid (C20:3), and Tetradecanedioic acid are closely associated with the prostaglandin-mediated regulation of platelet activation signaling pathways. The platelet activation signaling pathway plays an important role in inflammatory and immune responses, angiogenesis, and vasoconstrictive stretch [27,28,29,30,31] (Tables S3 and S4). In recent years, it has been found that prostaglandins have important functions in pregnancy, with a tendency to increase in the amniotic fluid as pregnancy progresses, but today’s studies do not clearly define the functions of prostaglandin metabolism in pregnancy [27,28]. Angiogenesis is the starting point and key to fetal development, and insufficient angiogenesis and limited steroid synthesis at the fetal–maternal interface have been found to be the main causes of early pregnancy loss in buffaloes [7,16,17]. The platelet activation signaling pathway and the complement and coagulation cascade signaling pathway are involved in placenta–fetal vascular tissue production and maternal immune response, which may be new targets for early pregnancy diagnosis in cattle [16,29]. In addition, differences in protein and metabolic profiles may be related to an active digestive system, which may be highly correlated with the living conditions and feed ratios of Chinese native yellow cattle. Endocrine disrupting chemicals (EDCs) strongly interfere with steroid hormone levels related to pregnancy maintenance [32]. For example, Kinetin 9-riboside, a plant hormone agonist detected in this experiment, has unknown functions during mammalian pregnancy, possibly due to significant expression differences caused by regional and dietary variations [33].

### 4.4. Limitations and Prospects of the Experiment

As previously mentioned, future studies with larger scale cohorts must, undoubtedly, back-up our research outcomes. Methodologically, the implementation of synchronized estrus protocols—employing gonadorelin for ovulation induction and cloprostenol sodium for luteolysis—introduced potential confounders. These hormonal interventions may have disrupted maternal endocrine homeostasis during early gestation, potentially compromising the specificity of the identified pregnancy-associated biomarkers [4,6]. Furthermore, the absence of standardized semen cryobanks for Chinese indigenous yellow cattle necessitated natural mating with native bulls to preserve the germplasm integrity, a protocol that inadvertently introduced variability in fertilization efficiency. Concurrently, emerging evidence suggests that dynamic shifts in vaginal microbiota could modulate early gestational physiology, introducing additional biological variability [34,35].

To overcome these limitations, we recommend three key improvements in subsequent studies: first, include more animals (especially controls) to confirm the results; second, combine proteomic and metabolomic analyses with microbiome studies to better understand hormonal–pregnancy interactions; and third, create standard breeding methods for these cattle, including semen cryopreservation and controlled artificial insemination.

## 5. Conclusions

This study found that the platelet activation signaling pathway and angiogenesis-related proteins were significantly upregulated during early pregnancy. Among them, FG showed significant differences, with FGB being highly upregulated and gradually increasing as pregnancy progressed. The consistent elevation of FGB in pregnant cows suggested its potential as a biomarker for early pregnancy detection, allowing for differentiation between pregnant and non-pregnant cows through protein analysis. Additionally, the differential metabolites 11-Deoxy prostaglandin F1α, Thromboxane B1, and Homo-Gamma-Linolenic Acid (C20:3) were involved in regulating the platelet activation signaling pathway. Together, these factors contributed to fetal–embryonic vascular tissue formation and the maternal immune response.

## Data Availability

The original contributions presented in this study are included in the article/Supplementary Materials. Further inquiries can be directed to the corresponding authors.

## Supplementary Materials

The following supporting information can be downloaded at: https://www.mdpi.com/article/10.3390/ani15091219/s1, Figure S1: Preliminary materials and methods; Figure S2: Analysis of serum identified proteins in early cattle pregnancy; Figure S3: Analysis of serum identified metabolite in early cattle pregnancy; Figure S4: The LC-MS/MS technique was used to validate and improve the reliability and stability of the data by enlarging the sample size within the group (5 cows) and increasing the 28-day samples; Table S1: Differential protein screening table; Table S2: Differential protein function search results; Table S3: Differential metabolite screening table; Table S4: Differential metabolites function search results. The references cited in the Supplementary Materials are listed in references [36,37,38,39,40,41,42,43,44,45,46,47,48,49,50,51,52,53,54,55,56,57,58].

## Abbreviations

4D-DIA	Four-dimensional data-independent acquisition
LC-MS-MS	Liquid chromatography–tandem mass spectrometry
FG	Fibrinogen alpha/beta/gamma chain
FGB	Fibrinogen beta chain
DAMs	Differentially abundant metabolites
DEPs	Differentially expressed proteins
