# Supplementary material for: The Platelet Activation Signaling Pathway Regulated by Fibrinogen and Homo-Gamma-Linolenic Acid (C20:3)-Associated Lipid Metabolism Is Involved in the Maintenance of Early Pregnancy in Chinese Native Yellow Cattle
Source: Animals (Basel). 2025 Apr 25;15(9):1219. doi: 10.3390/ani15091219 (PMC12071009; doi:10.3390/ani15091219)
Supplement: Supplementary file 1 [file animals-15-01219-s001.zip › animals-3515096-supplementary.pdf]

Platelet Activation Signaling Pathway Regulated by Fibrinogen and Homo-Gamma-Linolenic Acid (C20:3)-Associated Lipid Metabolism is Involved in the Maintenance of Early Pregnancy in Chinese Native Yellow Cattle

Miao Yu[∇a,b], Changzheng Du [∇a,b], Yabo Ma [a,b], Yuqin Ma [a,b], Pengfei Li [a,b], Xianguo Xie [a,b], Mengyuan Li [a,b], Xueyi Nie [a,b], Yueyang Liu [a,b], Yuxin Hou [a,b], Shenao Miao [a,b], Xinping Wang [a,c], Jinrui Xu* [a,b], Yi Yang* [a,b]

[∇] *Co-first Author*

[a] *School of Life Sciences, Ningxia University, Yinchuan, Ningxia 750021, China*

[b] *Key Laboratory of Ministry of Education for Conservation and Utilization of Special Biological Resources in the Western, Ningxia University, Yinchuan, Ningxia 750021, China*

[c] *College of Animal Science and Technology, Ningxia University, Yinchuan, Ningxia 750021, China*

Corresponding author: Jinrui Xu, Email: xujr@nxu.edu.cn

Yi Yang, Email: yangyi@nxu.edu.cn

TABLE OF CONTENTS

Table S1. Differential protein screening table.

Table S2. Differential protein function search results.

Table S3. Differential metabolite screening table.

Table S4. Differential metabolites function search results.

Figure S1. Preliminary materials and methods.

Figure S2. Analysis of serum identified proteins in early cattle pregnancy.

Figure S3. Analysis of serum identified metabolite in early cattle pregnancy.

Figure S4. The LC-MS/MS technique was used to validate and improve the reliability and stability of the data by enlarging the sample size within the group (5 cows) and increasing the 28-day samples.

4D-DIA proteomic analysis protocol

LC-MS/MS metabolites analysis protocol

Table S1. Differential protein screening table.

Protein	Description	Gene	GY_21.vs.GY_0 P-value	GY_21.vs.GY_0 log2FC	GY_21.vs.GY_0 UP.DOWN
A0A3Q1MP70	Glucose-6-phosphate isomerase	GPI	0	Inf	up
A0A3Q1MG04	Fibrinogen beta chain	FGB	0.003415	2.166736	up
F6QND5	Fibrinogen alpha chain	FGA	0.004334	1.452166	up
F1MVP0	ADAM metallopeptidase with thrombospondin type 1 motif 13	ADAMTS13	0.005649	-0.83595	down
Q1RMN9	C4b-binding protein alpha-like	LOC510860	0.008831	-2.40358	down
A7E3W2	Galectin-3-binding protein	LGALS3BP	0.012046	-0.86769	down
F6RBS0	GLI pathosis related 2	GLIPR2	0.012449	-1.88693	down
F1MGU7	Fibrinogen gamma-B chain	FGG	0.012479	2.208762	up
A0A6F8Z1X1	Glycosylation-dependent cell adhesion molecule 1	GlyCam1	0.016539	-1.20163	down
Q2HJB6	Procollagen C-endopeptidase enhancer	PCOLCE	0.017344	-0.96427	down
A0A140T881	Apolipoprotein E	APOE	0.027933	-0.27219	down
A0A452DI25	Hemopexin	HPX	0.030863	-1.82213	down
A0A3Q1LLU1	von Willebrand factor	VWF	0.04477	0.799173	up
P19034	Apolipoprotein C-II	APOC2	0.049415	2.485484	up

*Arrange in ascending order based on P-value.

Table S2. Differential protein function search results.

**Gene.**	**Function**	**References**
FGB<br>FGG<br>FGA	Fibrinogen, also known as Factor 1, consists of 21 pairs of polypeptide chains A peptide, Bβ and β, which play an important role in the immune system and coagulation processes. Clinical studies have shown that fibrinogen is significantly elevated in women during pregnancy, increasing the body's immune response.	[1, 2]
VWF<br>ADAMTS13	ADAMTS13 is a zinc metalloproteinase that specifically cleaves certain peptide bonds exposing VWF, a multimeric plasma glycoprotein that mediates platelet adhesion and aggregation. Studies have shown that mRNA transcripts of VWF increase in a time-dependent manner during mammalian pregnancy and promote angiogenesis.	[1, 3, 4]
HPX	Hemoglobin consists of four Hemopexin and one bead protein, while Hemopexin consists of four pyrrole subunits with a central iron ion. During pregnancy, maternal free hemoglobin is converted via the placenta into fetal hemoglobin, which is involved in physiological processes such as angiogenesis, iron metabolism, and immunomodulation.	[5, 6]
GlyCam1	Glycosylation-dependent cell adhesion molecule 1 (Gly-CAM-1) has different roles depending on where it is expressed. The breast, placenta and endometrium are among the organs that secrete this protein.	[7, 8]
LOC510860	C4BPA is involved in the complement pathway of the immune system in the circulation. It was shown that fetal C4BPA induced activation of atypical NF-κB pathway in the placenta may play an important role during full-term or preterm labor.	[9, 10]
LGALS3BP	Galectin-3-binding protein is involved in a variety of cellular processes, including cell adhesion, migration and immune responses. Studies have shown that LGALS6BP is actively transcriptionally expressed during early cattle pregnancy and is expected to be a potential marker for early cattle pregnancy 。	[11, 12]
PCOLCE	Procollagen C-endopeptidase enhancer (PCOLCE) is a key protein in collagen processing and a precursor of collagen. And protofibrillar collagen has a decisive role in fetal bone formation.	[13, 14]
APOE	Apolipoprotein E (APOE) is involved in the metabolism of lipids in the body, produced in large quantities in mammalian ovaries, regulated the production of steroid hormones, and its been shown to play an important role in lipid metabolism during pregnancy.	[15, 16]
APOC2	Apolipoproteins have complex physiologic functions in maternal plasma during pregnancy. It has been demonstrated that APOC2 has a tendency to increase in maternal plasma during pregnancy, especially during parturition.	[17]

Table S3. Differential metabolite screening table.

Compound_ID	Name	log2FC	P-value	VIP	Up.Down
Com_2418_pos	11-Deoxy prostaglandin F1α	1.563261	0.000296	1.945662	up
Com_2452_pos	KKK	3.848302	0.000961	1.922888	up
Com_10020_pos	12(S)-HETE	-1.62812	0.001013	1.934184	down
Com_7026_pos	(9cis)-Retinal	-1.85357	0.001383	1.92078	down
Com_2221_pos	HLK	2.437209	0.00148	1.908512	up
Com_9083_neg	Thromboxane B1	3.334588	0.001984	1.940496	up
Com_3937_pos	Pantothenic acid	-0.5032	0.002051	1.905229	down
Com_5632_pos	17alpha-Hydroxyprogesterone	-1.96233	0.002407	1.916278	down
Com_8908_neg	Kinetin 9-riboside	3.505599	0.002706	1.925861	up
Com_2531_pos	N-(4-butyl-2-methylphenyl)-N'-[4-(4-methylpiperazino) phenyl] urea	3.881262	0.003987	1.894334	up
Com_8936_neg	Lysopc 17:0	-0.57172	0.004331	1.864365	down
Com_2825_pos	THC	-3.36512	0.004828	1.871611	down
Com_8316_neg	FAHFA (17:2/8:0)	-1.39962	0.005592	1.868842	down
Com_5695_pos	fentanyl-d5	1.092271	0.006543	1.902624	up
Com_6300_pos	Prostaglandin G2	0.922198	0.006986	1.844852	up
Com_9006_neg	O1-(4-chlorobenzoyl)-4-nitrobenzene-1-carbohydroximamide	0.334249	0.007257	1.851688	up
Com_14138_pos	L-Saccharopine	1.963268	0.009288	1.903706	up
Com_9075_neg	Tetradecanedioic acid	0.760047	0.010264	1.807044	up
Com_2829_pos	β-Cortolone	-2.55597	0.010574	1.817485	down
Com_2896_pos	11(Z),14(Z),17(Z)-Eicosatrienoic acid	3.696078	0.010937	1.881512	up
Com_3768_pos	PC (16:2/17:2)	-1.25439	0.011205	1.880546	down
Com_6178_pos	3-(3,4,5-trimethoxyphenyl) propanoic acid	0.753482	0.012404	1.789402	up
Com_7782_pos	1,2-dihydroxyheptadec-16-yn-4-yl acetate	0.747695	0.012856	1.88877	up
Com_8314_neg	FAHFA (16:0/6:0)	0.711031	0.013618	1.796083	up
Com_608_pos	SM (d14:0/20:0)	-0.6804	0.013808	1.834543	down
Com_3587_pos	8Z,11Z,14Z-Eicosatrienoic acid	2.51835	0.015179	1.781226	up
Com_8700_neg	2-Phenylglycine	0.544039	0.015716	1.76626	up
Com_8537_neg	(±)17(18)-DiHETE	-0.68254	0.016383	1.806538	down

Com_6279_pos	Homo-Gamma-Linolenic Acid (C20:3)	1.781054	0.018121	1.757327	up
Com_3364_pos	2,5-bis(4-hydroxy-3-methoxyphenyl)-3,4-dimethyloxolan-3-ol	3.071793	0.018404	1.895558	up
Com_3653_pos	Desoxycortone	-2.18792	0.019852	1.739927	down
Com_6995_pos	Andrographolide	-1.64863	0.020539	1.806238	down
Com_8384_neg	LPC 19:1	-0.73456	0.020638	1.812651	down
Com_849_pos	Ecgonine	1.007709	0.022758	1.728465	up
Com_6223_pos	ACar 24:0	2.265631	0.023409	1.729517	up
Com_7479_pos	Ergosta-5,7,9(11),22-Tetraen-3-beta-Ol	-1.63047	0.024616	1.828091	down
Com_8539_neg	(±)18-HEPE	-2.12251	0.025507	1.791967	down
Com_4109_pos	cis-gondoic acid	2.297047	0.028063	1.809993	up
Com_2972_pos	16,16-Dimethyl prostaglandin A1	0.488038	0.028878	1.727584	up
Com_8312_neg	FAHFA (14:0/6:0)	0.609664	0.030201	1.692845	up
Com_11079_pos	L-Palmitoylcarnitine	0.932807	0.030981	1.726419	up
Com_11889_pos	Vitamin A	-0.92058	0.031192	1.748095	down
Com_8410_neg	LPE 17:0	-0.47484	0.03157	1.720008	down
Com_341_pos	DL-Carnitine	-0.68393	0.033461	1.671018	down
Com_8942_neg	Lysope 16:0	-1.13567	0.03375	1.673217	down
Com_8562_neg	(3-Methoxy-4-hydroxyphenyl) ethylene glycol sulfate	2.948564	0.036974	1.746693	up
Com_8696_neg	2-Isopropylmalate	0.509159	0.037158	1.667757	up
Com_5298_pos	All-Trans-13,14-Dihydroretinol	1.13919	0.042695	1.73436	up
Com_8910_neg	L-Adrenaline	1.500285	0.043746	1.650197	up
Com_8614_neg	13,14-dihydro-15-keto-tetranor Prostaglandin D2	0.579299	0.045409	1.683223	up
Com_209_pos	SM (d20:1/15:0)	-0.6363	0.045575	1.699616	down
Com_8601_neg	11-keto Testosterone (CRM)	-1.78526	0.047334	1.790363	down
Com_3404_pos	Sedanolide	1.392895	0.04955	1.683792	up

*Arrange in ascending order based on P-value.

Table S4. Differential metabolites function search results.

Name	Function	References
Homo-Gamma-Linolenic Acid (C20:3)<br>Tetradecanedioic acid<br>11-Deoxy prostaglandin F1α<br>Thromboxane B1	Gamma-linolenic acid is a polyunsaturated fatty acid found in milk and plants , and is an important dietary supplement; it can be converted into long-chain fatty acid-like organic compounds in the body, and can be further converted into lipids such as Prostaglandin and Thromboxane.11-Deoxyprostaglandin F1 alpha is a thromboxane A2 receptor agonist, while Thromboxane B1 is a metabolite of thromboxane A2. Prostaglandins are important in mammalian pregnancy and are involved in platelet activation and aggregation, vasoconstriction and inflammatory responses in vivo, and it has been found that prostaglandins increase as pregnancy progresses.	[18-21]
L-Palmitoylcarnitine<br>DL-Carnitine	L-Palmitoylcarnitine is an ester derivative of DL-Carnitine involved in fatty acid β-oxidation synthesised via carnitine palmitoyltransferase I (CPT1), which is antagonistic to L-carnitine. The expression of L-Palmitoylcarnitine was found to be significantly increased during early mammalian pregnancy, and the reduction in plasma L-carnitine in pregnant cows was mainly attributed to placental uptake.	[22-24]
Kinetin 9-riboside	Kinetin 9-riboside is a phytohormone that is absorbed into the body's circulation through the mammalian intestine and has anti-inflammatory properties and promotes cell growth.	[25, 26]
(9cis)-Retinal	(9cis)-Retinal is also known as retinaldehyde, a natural metabolite of vitamin A. And it is also known as retinoid. And it plays an important role in the embryonic visual system. A study on the addition of (9cis)-Retinal to retinal organoids inducing the development of pluripotent stem cell lines showed that it accelerated optic stem photoreceptor differentiation in organoid cultures.	[27, 28]
11-keto Testosterone (CRM)	11-keto Testosterone (CRM), an endogenous active androgen, is a potent androgen receptor (AR) agonist, which is particularly important during fetal development.	[29, 30]
β-Cortolone	β-Cortolone is a synthetic glucocorticoid steroid with anti-inflammatory and immunosuppressive properties, which is absorbed into the placenta during mammalian pregnancy and is involved in fetal development.	[31, 32]
Andrographolide	Andrographolide is a plant active compound that is absorbed through the intestines into the body's circulation and has anti-inflammatory and immune system enhancing properties.	[33, 34]

Figure S1. Preliminary materials and methods.

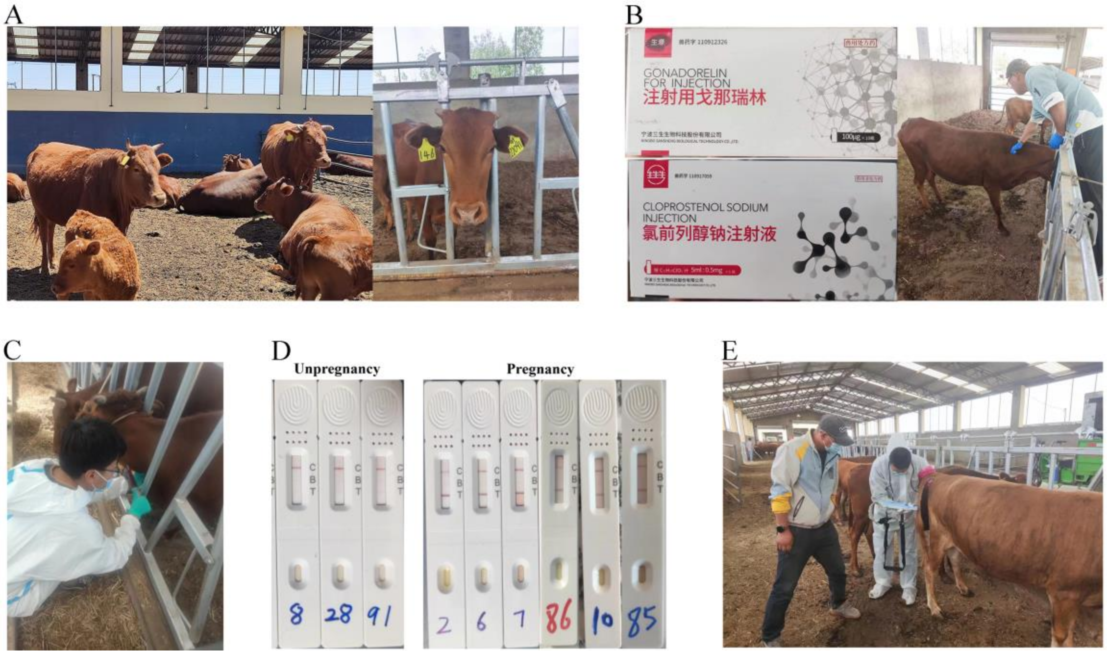

*All figures taken from the experimental site. A, experimental animals. B, simultaneous estrus and natural mating in non-pregnant cows. C, collection and preservation of serum. D, cattle early pregnancy tests and ultrasound to diagnose pregnancy status.

Figure S2. Analysis of serum identified proteins in early cattle pregnancy.

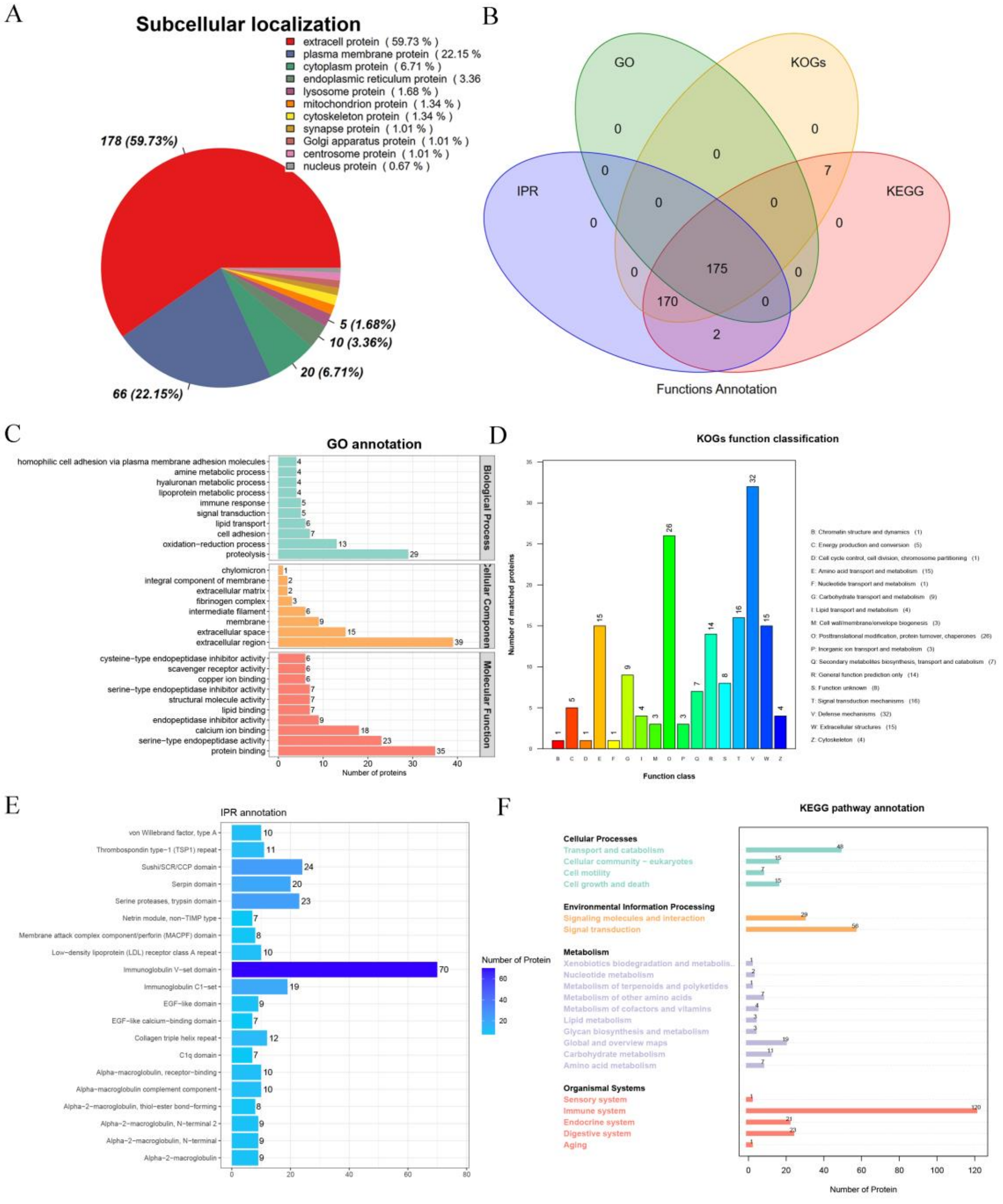

***A**, subcellular localization annotation results pie chart. B, functional annotation results, each circle in the graph represents an annotation result from a database, and the overlapping parts represent proteins annotated by multiple databases. C, GO annotation results bar chart, showing only the top 10 results in each major category, with the horizontal axis representing the number of proteins and the vertical axis representing the annotated GO entries. D, KOG annotation results

bar chart, with the horizontal axis representing the functional classification of annotations and the vertical axis representing the number of proteins annotated to the corresponding function. E, Structural domain annotation results bar chart (IPR), with the horizontal axis representing the number of proteins and the vertical axis representing the annotated IPR entries. F, KEGG annotation results bar chart, with the horizontal axis representing the number of proteins and the vertical axis representing the annotated KEGG entries.

Figure S3. Analysis of serum identified metabolite in early cattle pregnancy.

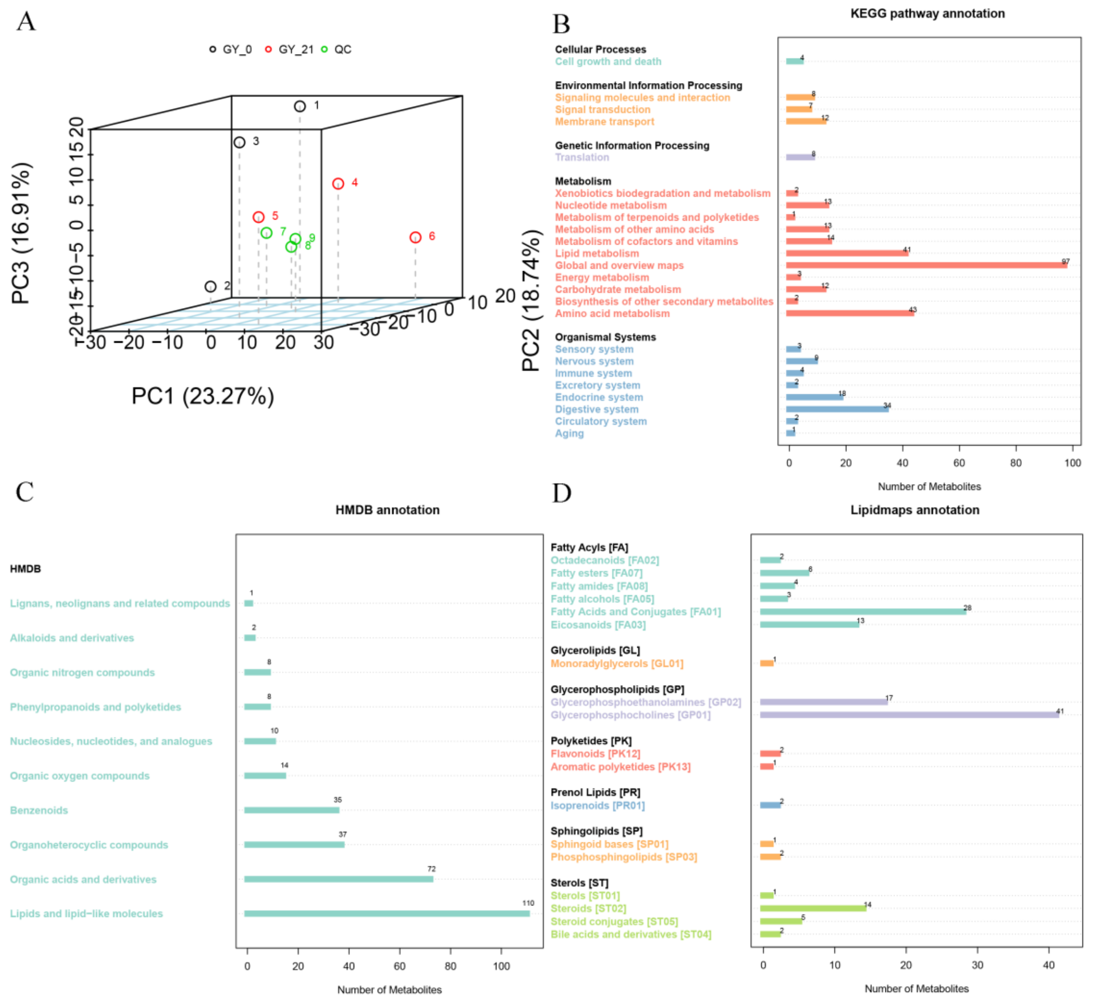

***A**, Principal Component Analysis (PCA) of the overall samples, where PC1, PC2, and PC3 in the graph represent the scores of the first, second, and third ranked principal components respectively. Different colored dots represent samples from different experimental groups. B, KEGG pathway annotation, with the horizontal axis representing the number of metabolites and the vertical axis representing the annotated KEGG pathways. C, HMDB classification annotation, with the horizontal axis representing the number of metabolites and the vertical axis representing the annotated HMDB classifications. D, LIPID MAPS classification annotation, with the horizontal axis representing the number of metabolites and the vertical axis representing the annotated LIPID MAPS lipid classifications.

Figure S4. The LC-MS/MS technique was used to validate and improve the reliability and stability of the data by enlarging the sample size within the group (5 cows) and increasing the 28-day samples.

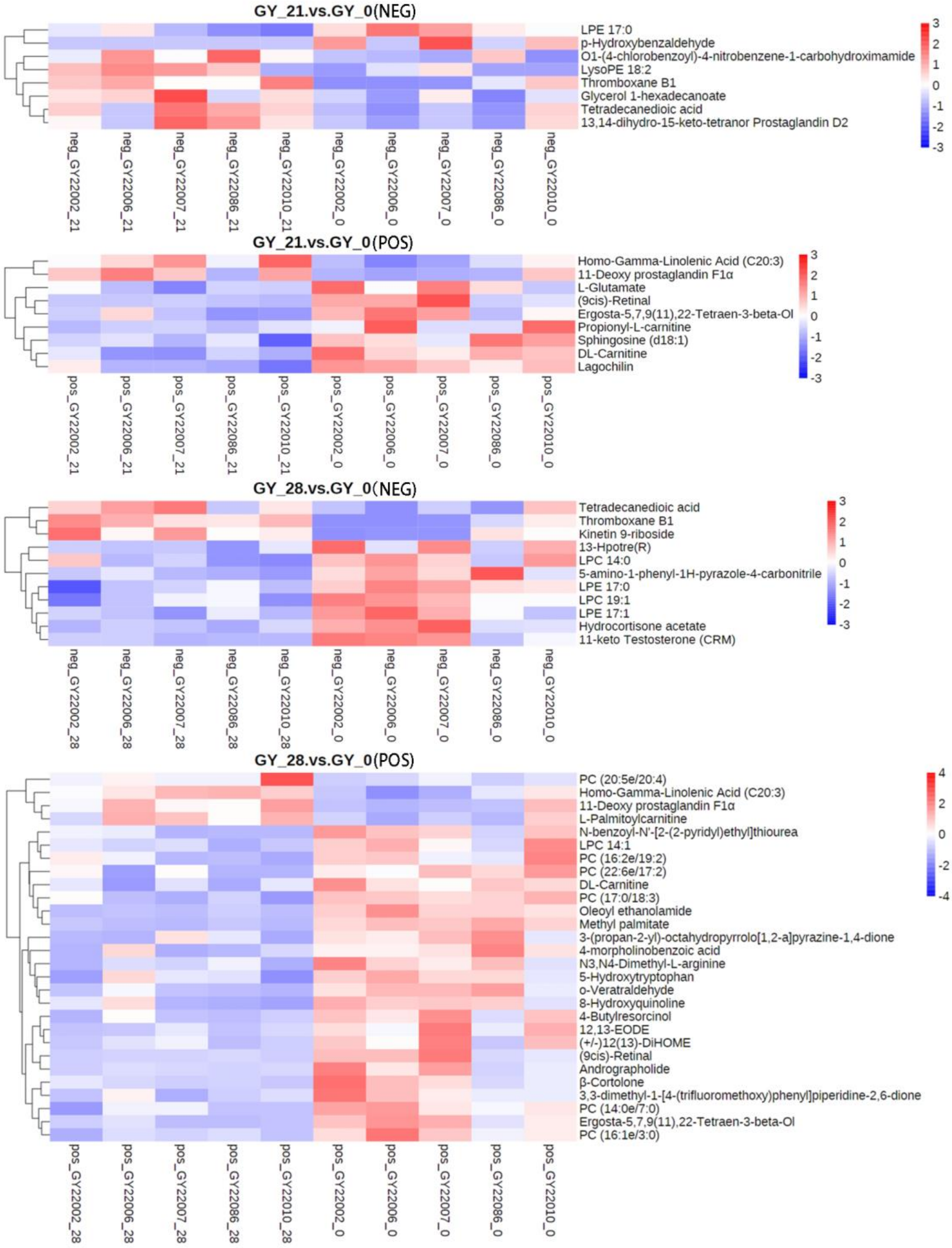

*clustering heatmap, the horizontal axis represents sample clustering, the vertical axis represents

metabolites clustering, with shorter clustering branches representing higher similarity. NEG, negative ion metabolite; POS, positive ion metabolite.

4D-DIA proteomic analysis

Sample Preparation:

Protein Dissolution: Take the protein sample and add DB protein lysis buffer (8M urea, 100 mM TEAB, pH 8.5) to a final volume of 100 μL.

Enzymatic Digestion: Add trypsin and 100 mM TEAB buffer, mix well, and incubate at 37°C for 4 hours. Then add trypsin and CaCl2 for overnight digestion.

pH Adjustment and Desalting

pH Adjustment: Add formic acid to adjust the pH to less than 3.

Centrifugation: Centrifuge at 12000 g for 5 minutes at room temperature.

Desalting: Pass the supernatant slowly through a C18 desalting column.

Washing: Wash the column three times with a cleaning solution made of 0.1% formic acid and 3% acetonitrile.

Elution: Elute with a solution containing 0.1% formic acid and 70% acetonitrile. Collect the eluate and lyophilize.

DDA Library Construction:

Fractionation:

Preparation of Mobile Phases: Prepare mobile phase A (2% acetonitrile, 98% water, pH adjusted to 10 with ammonia) and mobile phase B (98% acetonitrile, 2% water, pH adjusted to 10 with ammonia).

Dissolution and Centrifugation: Dissolve the lyophilized powder in mobile phase A and centrifuge at 12000 g for 10 minutes at room temperature.

HPLC Fractionation: Use a Waters BEH C18 column (4.6×250 mm, 5μm) with a column temperature of 45°C. Collect fractions every minute and combine into 4 or 6 fractions (see Tables 3-3 and 3-4). Lyophilize and dissolve each fraction in 0.1% formic acid.

DDA Mass Detection:

Preparation of Mobile Phases: Prepare mobile phase A (100% water, 0.1% formic acid) and mobile phase B (80% acetonitrile, 0.1% formic acid).

Sample Injection: Take 4 μg of the supernatant and add 0.8 μL of iRT internal standard. Inject half of the volume into the EASY-nLCTM 1200 UHPLC system.

Chromatography Conditions: Use a homemade trap column (4.5 cm × 75 μm, 3 μm) and an analytical column (15 cm × 150 μm, 1.9 μm). Set the chromatography conditions according to Table 3-5.

Mass Spectrometry Conditions: Use a Q ExactiveTM HF-X mass spectrometer with a Nanospray Flex™ ESI ion source. Set the ion spray voltage to 2.1 kV and the ion transfer tube temperature to 320°C. Use DDA mode for data acquisition with a full scan range of m/z 350 to 1500. The resolution of the first-order mass spectrum is 120000 (at 200 m/z). The maximum capacity of the C-trap is 3×10^6, and the maximum injection time is 80 ms. Select the top 40 most intense ions for HCD fragmentation with a resolution of 15000 (at 200 m/z). The maximum capacity of the C-trap is 5×10^4, and the maximum injection time is 45 ms. Set the collision energy to 27% and the threshold intensity to 1.1×10^4. Use a dynamic exclusion time of 20 seconds to obtain the raw mass spectrometry data (.raw) for constructing the DDA library.

DIA Mass Detection:

nanoElute-Tims TOF pro2

Preparation of Mobile Phases: Prepare mobile phase A (100% water, 0.1% formic acid) and mobile phase B (100% acetonitrile, 0.1% formic acid).

Sample Dissolution and Centrifugation: Dissolve the lyophilized powder in 10 μL of mobile phase A and centrifuge at 14000 g for 20 minutes at 4°C.

Sample Injection: Inject 200 ng of the supernatant into the LC-MS system.

Chromatography Conditions: Use a nanoElute UHPLC system with an analytical column (25 cm × 75 μm, 1.6 μm). Set the chromatography conditions according to Table 3-6.

Mass Spectrometry Conditions: Use a Tims TOF pro2 mass spectrometer with a Captive Spray ion source. Set the ion spray voltage to 1.5 kV. The full scan range is m/z 100-1700. Set the ramp time to 100 ms and the lock duty cycle to 100%. Use PASEF with 10 MS/MS scans (total cycle time 1.17 seconds). Set the ion intensity threshold to 2500 and the scheduling target intensity to 20000. Generate raw mass spectrometry data (.d) for constructing the DDA library.

Mass Spectrometry Data Analysis

Protein Identification and Quantification

Database Search: Use Spectronaut-Pulsar (Biognosys) software to search the DDA data. Set the precursor ion mass tolerance to 10 ppm and the fragment ion mass tolerance to 0.02 Da. Fixed modifications include cysteine alkylation, and variable modifications include methionine oxidation and N-terminal acetylation. Allow up to 2 missed cleavage sites.

Data Filtering: Filter the search results using Spectronaut-Pulsar software. Retain peptides with a confidence level of 99% or higher (PSMs) and proteins with at least one unique peptide. Remove

peptides and proteins with a false discovery rate (FDR) greater than 1%.

Quantitative Analysis: Import DIA data into Spectronaut software and use the Pulsar-built DDA library to analyze ion pairs and calculate peak areas for peptide identification and quantification. Use iRT to correct retention times and set the precursor ion Qvalue cutoff to 0.01. Use t-tests to statistically analyze protein quantification results and define differentially expressed proteins (DEPs) based on significant differences between experimental and control groups.

Functional Analysis of Proteins and DEPs

Functional Annotation: Use InterProScan software for GO and IPR functional annotation (including Pfam, PRINTS, ProDom, SMART, ProSite, PANTHER databases). Perform COG and KEGG annotations for functional protein family and pathway analysis.

Enrichment Analysis: Conduct volcano plot analysis, clustering heatmap analysis, and GO, IPR, and KEGG pathway enrichment analysis on DEPs. Use STRING DB software to predict potential protein-protein interactions.

LC-MS/MS metabolites analysis

Metabolite Extraction Procedure:

Place 100 μL of the sample into an EP tube and add 400 μL of 80% methanol aqueous solution to the tube. Vortex mix, then incubate on ice for 5 minutes, followed by centrifugation at 15000 g for 20 minutes at 4°C. Take a certain amount of the supernatant and dilute it with water to achieve a methanol concentration of 53%. Centrifuge again at 15000 g for 20 minutes at 4°C, collect the supernatant, and proceed with analysis using LC-MS.

QC Sample: Mix equal volumes of liquid from each experimental sample to create the QC sample.

Blank Sample: Use a 53% methanol aqueous solution, and process it in the same manner as the experimental samples.

Instrument parameters:

Column: Hypersil Gold column (C18)

Column Temperature: 40°C

Flow Rate: 0.2 mL/min

Mobile Phase A in Positive Mode: 0.1% formic acid

Mobile Phase B in Positive Mode: Methanol

Mobile Phase A in Negative Mode: 5 mM ammonium acetate, pH 9.0

Mobile Phase B in Negative Mode: Methanol

The chromatographic gradient elution program is as follows:

The gradient elution begins with 98% mobile phase A and 2% mobile phase B, held constant for the first 1.5 minutes. From 1.5 to 3 minutes, a linear gradient shifts the composition to 15% A and

85% B. The gradient continues to transition, reaching 0% A and 100% B by 10 minutes. Immediately after, at 10.1 minutes, the composition returns to the initial condition of 98% A and 2% B, which is maintained until 12 minutes to allow for column re-equilibration.

Mass Spectrometry Conditions:

Scan Range: m/z 100-1500

ESI Source Settings:

Spray Voltage: 3.5 kV

Sheath Gas Flow Rate: 35 psi

Aux Gas Flow Rate: 10 L/min

Capillary Temp: 320℃

S-lens RF Level: 60

Aux Gas Heater Temp: 350℃

Polarity: Positive, Negative

MS/MS Scanning: Data-dependent scans

Data Preprocessing and Metabolite Identification:

Utilize CD3.1 search library software to import raw data files (.raw) and perform preliminary screening of retention time, mass-to-charge ratio, and other parameters for each compound. Set retention time deviation to 0.2 min and mass deviation to 5 ppm for peak alignment in each sample to enhance identification accuracy. Extract peaks based on the set mass deviation of 5 ppm, signal intensity deviation of 30%, signal-to-noise ratio of 3, and minimum signal intensity, and perform quantitative analysis of peak areas. Integrate target ion information and predict molecular formulas by analyzing molecular ion peaks and fragment ions. Compare the predicted results with

mzCloud, mzVault, and Masslist databases, and use blank samples to eliminate background ions. Normalize the original quantitative results using the following formula: divide the original quantitative value of the sample by (the total quantitative value of metabolites in the sample divided by the total quantitative value of metabolites in QC1 sample) to obtain relative peak areas. During this process, remove compounds with a relative peak area coefficient of variation greater than 30% in QC samples to obtain the final metabolite identification and relative quantitative results. Data processing is based on the Linux operating system (CentOS version 6.6) and software R and Python.

REFERENCES

1. Tanaka, K.A., et al., *Elevated fibrinogen, von Willebrand factor, and Factor VIII confer resistance to dilutional coagulopathy and activated protein C in normal pregnant women.* Br J Anaesth, 2019. **122**(6): p. 751-759.
2. Usta, C.S., et al., *Does the fibrinogen/albumin ratio predict the prognosis of pregnancies with abortus imminens?* Saudi Med J, 2021. **42**(3): p. 255-263.
3. Kumar, S., et al., *Expression and functional role of bone morphogenetic proteins (BMPs) in placenta during different stages of pregnancy in water buffalo (Bubalus bubalis).* Gen Comp Endocrinol, 2020. **285**: p. 113249.
4. Stepanian, A., et al., *Von Willebrand factor and ADAMTS13: a candidate couple for preeclampsia pathophysiology.* Arterioscler Thromb Vasc Biol, 2011. **31**(7): p. 1703-9.
5. Cao, C., et al., *Placental heme receptor LRP1 correlates with the heme exporter FLVCR1 and neonatal iron status.* Reproduction, 2014. **148**(3): p. 295-302.
6. Bellos, I., et al., *The role of hemoglobin degradation pathway in preeclampsia: A systematic review and meta-analysis.* Placenta, 2020. **92**: p. 9-16.
7. Valk-Weeber, R.L., et al., *Variations in N-linked glycosylation of glycosylation-dependent cell adhesion molecule 1 (GlyCAM-1) whey protein: Intercow differences and dietary effects.* Journal of dairy science, 2021. **104**(4): p. 5056-5068.
8. Stenhouse, C., et al., *Insights into the regulation of implantation and placentation in humans, rodents, sheep, and pigs.* Recent Advances in Animal Nutrition and Metabolism, 2022: p. 25-48.
9. Ithier, M.C., et al., *Fetal lung C4BPA induces p100 processing in human placenta.* Scientific reports, 2019. **9**(1): p. 1-10.
10. McElroy, J.J., et al., *Maternal coding variants in complement receptor 1 and spontaneous idiopathic preterm birth.* Hum Genet, 2013. **132**(8): p. 935-42.
11. Sharma, P., et al., *Investigation of conceptus stimulated gene expression in buffalo peripheral blood mononuclear cells as potential diagnostic markers of early pregnancy.* J Dairy Res, 2023. **90**(2): p. 142-145.
12. Cho, S.H., et al., *Lgals3bp suppresses colon inflammation and tumorigenesis through the downregulation of TAK1-NF-κB signaling.* Cell Death Discov, 2021. **7**(1): p. 65.
13. Hulmes, D.J.S., *Roles of the procollagen C-propeptides in health and disease.* Essays Biochem, 2019. **63**(3): p. 313-323.
14. Chen, Y., et al., *Type-I collagen produced by distinct fibroblast lineages reveals specific function during embryogenesis and Osteogenesis Imperfecta.* Nat Commun, 2021. **12**(1): p. 7199.
15. Oriá, R.B., et al., *Apolipoprotein E Effects on Mammalian Ovarian Steroidogenesis and Human Fertility.* Trends Endocrinol Metab, 2020. **31**(11): p. 872-883.
16. Xie, Y., et al., *Early Gestational Blood Markers to Predict Preeclampsia Complicating Gestational Diabetes Mellitus.* Diabetes Metab Syndr Obes, 2023. **16**: p. 1493-1503.
17. Flood-Nichols, S.K., et al., *Longitudinal analysis of maternal plasma apolipoproteins in pregnancy: a targeted proteomics approach.* Mol Cell Proteomics, 2013. **12**(1): p. 55-64.
18. Wood, E.M., K.K. Hornaday, and D.M. Slater, *Prostaglandins in biofluids in pregnancy and labour: A systematic review.* PLoS One, 2021. **16**(11): p. e0260115.

19. Pai, C.H., et al., *Lack of Thromboxane Synthase Prevents Hypertension and Fetal Growth Restriction after High Salt Treatment during Pregnancy.* PLoS One, 2016. **11**(3): p. e0151617.
20. Ye, X., H. Diao, and J. Chun, *11-deoxy prostaglandin F2α, a thromboxane A2 receptor agonist, partially alleviates embryo crowding in Lpar3(−/−) females.* Fertility and Sterility, 2012. **97**(3): p. 757-763.
21. Sergeant, S., E. Rahbar, and F.H. Chilton, *Gamma-linolenic acid, Dihommo-gamma linolenic, Eicosanoids and Inflammatory Processes.* European Journal of Pharmacology, 2016. **785**: p. 77-86.
22. Mutomba, M.C., et al., *Regulation of the activity of caspases by L-carnitine and palmitoylcarnitine.* FEBS Letters, 2000. **478**(1): p. 19-25.
23. Dong, R., et al., *Studies on Novel Diagnostic and Predictive Biomarkers of Intrahepatic Cholestasis of Pregnancy Through Metabolomics and Proteomics.* Front Immunol, 2021. **12**: p. 733225.
24. Bai, M., et al., *Maternal Plasma l-Carnitine Reduction During Pregnancy Is Mainly Attributed to OCTN2-Mediated Placental Uptake and Does Not Result in Maternal Hepatic Fatty Acid β-Oxidation Decline.* Drug Metab Dispos, 2019. **47**(6): p. 582-591.
25. Kim, S.W., et al., *Phytohormones: Multifunctional nutraceuticals against metabolic syndrome and comorbid diseases*. Biochem Pharmacol, 2020. **175**: p. 113866.
26. Neumann, K.-H., et al., *Phytohormones and growth regulators.* Plant Cell and Tissue Culture–A Tool in Biotechnology: Basics and Application, 2020: p. 309-319.
27. Kaya, K.D., et al., *Transcriptome-based molecular staging of human stem cell-derived retinal organoids uncovers accelerated photoreceptor differentiation by 9-cis retinal.* Mol Vis, 2019. **25**: p. 663-678.
28. Kelley, R.A., et al., *Accelerated Development of Rod Photoreceptors in Retinal Organoids Derived from Human Pluripotent Stem Cells by Supplementation with 9-cis Retinal.* STAR Protoc, 2020. **1**(1).
29. Imamichi, Y., et al., *11-Ketotestosterone Is a Major Androgen Produced in Human Gonads.* The Journal of Clinical Endocrinology & Metabolism, 2016. **101**(10): p. 3582-3591.
30. Turcu, A.F., et al., *11-Oxygenated androgens in health and disease.* Nature Reviews Endocrinology, 2020. **16**(5): p. 284-296.
31. Wang, R., et al., *Characterizing the steroidal milieu in amniotic fluid of mid-gestation: A GC-MS study.* The Journal of Steroid Biochemistry and Molecular Biology, 2019. **193**: p. 105412.
32. Stoye, D.Q., et al., *Maternal Glucocorticoid Metabolism Across Pregnancy: A Potential Mechanism Underlying Fetal Glucocorticoid Exposure.* The Journal of Clinical Endocrinology & Metabolism, 2020. **105**(3): p. e782-e790.
33. Zeng, B., et al., *Andrographolide: A review of its pharmacology, pharmacokinetics, toxicity and clinical trials and pharmaceutical researches.* Phytother Res, 2022. **36**(1): p. 336-364.
34. Li, X., et al., *Andrographolide, a natural anti-inflammatory agent: An Update.* Front Pharmacol, 2022. **13**: p. 920435.
